# No Fabry Disease in Patients Presenting with Isolated Small Fiber Neuropathy

**DOI:** 10.1371/journal.pone.0148316

**Published:** 2016-02-11

**Authors:** Bianca T. A. de Greef, Janneke G. J. Hoeijmakers, Emma E. Wolters, Hubertus J. M. Smeets, Arthur van den Wijngaard, Ingemar S. J. Merkies, Catharina G. Faber, Monique M. Gerrits

**Affiliations:** 1 Department of Neurology, School of Mental Health and Neuroscience, Maastricht University Medical Center, Maastricht, The Netherlands; 2 Department of Clinical Genetics, Maastricht University Medical Center, Maastricht, The Netherlands; 3 Department of Neurology, Spaarne hospital, Hoofddorp, The Netherlands; Baylor Research Institute, UNITED STATES

## Abstract

**Objective:**

Screening for Fabry disease in patients with small fiber neuropathy has been suggested, especially since Fabry disease is potentially treatable. However, the diagnostic yield of testing for Fabry disease in isolated small fiber neuropathy patients has never been systematically investigated. Our aim is to determine the presence of Fabry disease in patients with small fiber neuropathy.

**Methods:**

Patients referred to our institute, who met the criteria for isolated small fiber neuropathy were tested for Fabry disease by measurement of alpha-Galactosidase A activity in blood, lysosomal globotriaosylsphingosine in urine and analysis on possible GLA gene mutations.

**Results:**

725 patients diagnosed with small fiber neuropathy were screened for Fabry disease. No skin abnormalities were seen except for redness of the hands or feet in 30.9% of the patients. Alfa-Galactosidase A activity was tested in all 725 patients and showed diminished activity in eight patients. Lysosomal globotriaosylsphingosine was examined in 509 patients and was normal in all tested individuals. Screening of GLA for mutations was performed for 440 patients, including those with diminished α-Galactosidase A activity. Thirteen patients showed a GLA gene variant. One likely pathogenic variant was found in a female patient. The diagnosis Fabry disease could not be confirmed over time in this patient. Eventually none of the patients were diagnosed with Fabry disease.

**Conclusions:**

In patients with isolated small fiber neuropathy, and no other signs compatible with Fabry disease, the diagnostic yield of testing for Fabry disease is extremely low. Testing for Fabry disease should be considered only in cases with additional characteristics, such as childhood onset, cardiovascular disease, renal failure, or typical skin lesions.

## Introduction

Small fiber neuropathy (SFN) is a disorder of the thinly myelinated Aδ-fibers and unmyelinated C-fibers. These fibers are responsible for the sensation of temperature and pain and regulate a great deal of the autonomic nervous system. As a result, SFN is clinically characterized by neuropathic pain and autonomic symptoms [1, 2]. Patients with SFN experience different types of pain mainly described as a burning sensation, itching, prickling, or shooting pains. These symptoms usually occur in a symmetrical length-dependent pattern [3], but non-length dependent patterns have been described [4]. SFN is not a rare condition; a recent study showed a minimum prevalence of 53/100.000 [5]. Many diseases can underlie small nerve fiber damage, such as diabetes or impaired glucose tolerance, HIV infection, immune-mediated disorders (e.g. Sjögren syndrome and sarcoidosis) [2], and hereditary disorders (e.g. sodium channel gene mutations) [6–8]. Diabetes mellitus is considered one of the most common causes of SFN. Despite thorough investigations, an underlying cause cannot be identified in 38–48% of the patients [5, 9].

One of the possible causes of SFN is Fabry disease (FD) [10–12]. FD is an X-linked glycolipid storage disease due to a mutation in the GLA gene. This causes an absent or diminished activity of the lysosomal enzyme alpha-Galactosidase A (α-Gal A), leading to accumulation of globotriaosylceramide (lyso-GB3) in different cell types and subsequently to severe multi-system disease [13, 14]. The involvement of different cell types leads to various clinical manifestations, such as cardiovascular disease, renal failure, and skin lesions. The accumulation of lyso-GB3 also occurs in neurons, which causes neurological symptoms. The symptoms may develop in different periods of the patient’s life. Shooting pain and discomfort in the hands and feet, which are triggered by heat, exercise, and stress, are considered the most common symptoms that patient experience in early childhood and adolescence. About 70% of children and adolescents with classical FD develop pain in hands and feet [15]. These neuropathic pain symptoms can become more generalized over time [16]. However, it is not clear how often FD is the underlying cause in patients presenting with SFN.

The relationship between FD and small nerve fiber dysfunction has been described before [10–14]. The prevalence of SFN cases that are caused by FD is not well known. However, it is important to identify patients with FD, because it is a treatable disorder. Treatment with enzyme replacement therapy (ERT) may improve the quality of life and prevent serious and potential life-threatening complications [17–20]. Early ERT has been suggested to improve small nerve fiber function [14, 21, 22], although these findings could be not validated by others [23].

The aim of our study is to investigate the prevalence of FD in a well-defined cohort of patients diagnosed with SFN and related costs to this diagnostic testing.

## Methods

### Patients

We included all consecutive patients referred for possible SFN to the Maastricht University Medical Center (Maastricht UMC+) between August 2006 and April 2015. The diagnosis SFN was made if patients fulfilled the criteria for SFN as described earlier [2, 24]. These criteria include the presence of ≥ 2 typical symptoms for SFN not otherwise explained, no signs of large fiber involvement, and reduced intraepidermal nerve fiber density [25] and/or abnormal temperature thresholds in quantitative sensory testing [26, 27].

Screening for FD was performed in all patients with SFN as described in the following.

### Biochemical assessments and DNA analysis for FD

Presence of FD was examined through measurement of α-Gal A activity in leukocytes [28], lyso-GB3 excretion in urine [29] and screening of the GLA gene for mutations. α-Gal A enzyme activity is considered normal between 30 and 180 mmol/L and diminished <30 mmol/L.

Lyso-GB3 excretion is considered abnormal >0 nmol/mmol creatinine [29]. The measurement of lyso-GB3 excretion in urine was incorporated in our workflow for SFN patients from April 2012. Variants in the coding and intermediate flanking regions of the GLA gene were classified according to the Practice Guidelines for the Evaluation of Pathogenicity and the Reporting of Sequence Variant in clinical Molecular Genetics [30]. Class 1 and 2 variants are considered non-pathogenic. Class 3 variants have an uncertain pathogenicity. Class 4 variants are likely to be pathogenic and class 5 variants are certain pathogenic [30].

**Fig 1:**
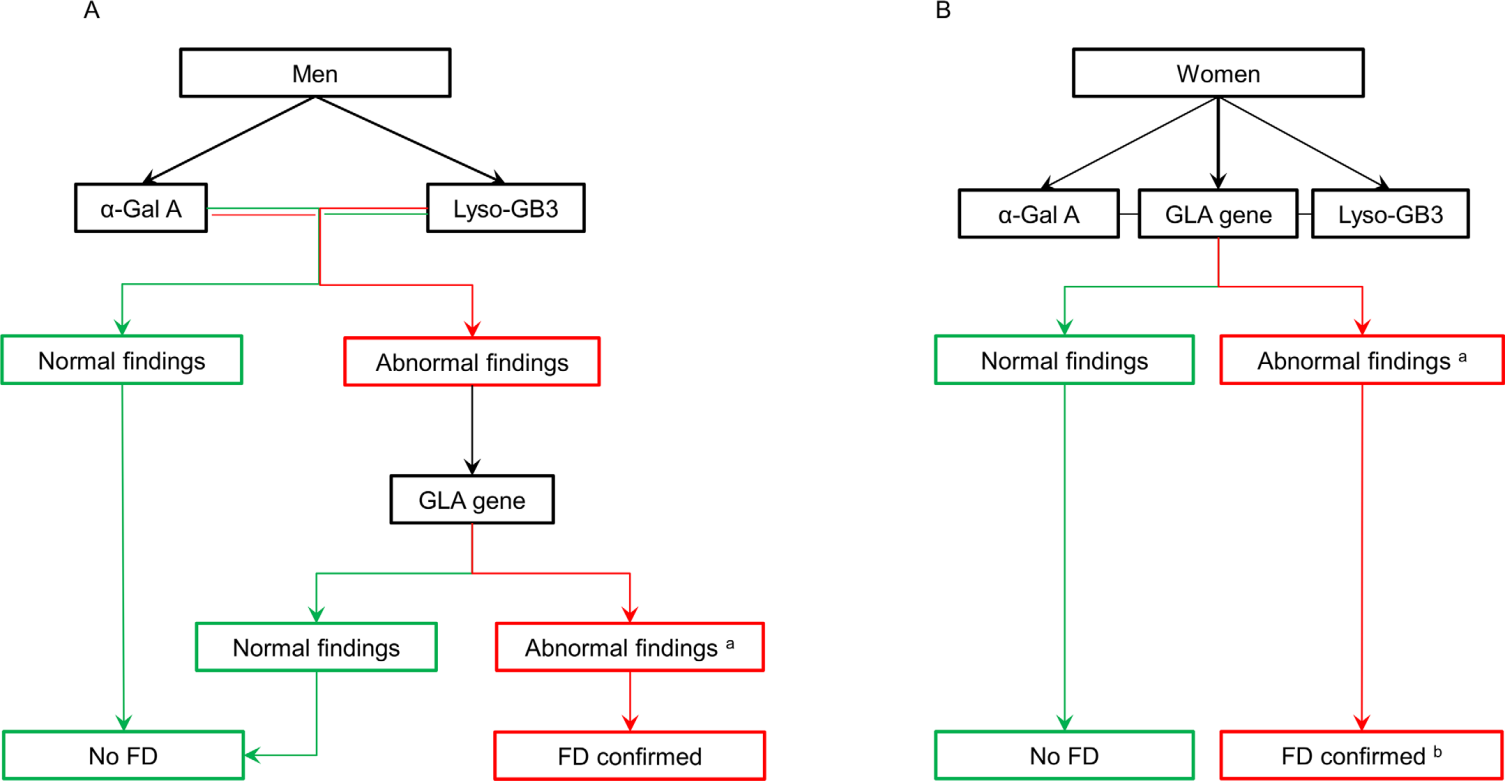
Diagnostic algorithm for confirming Fabry disease in SFN patients. (A) Diagnostic algorithm for men. (B) Diagnostic algorithm for women. α-Gal A: α-galactosidase A, FD: Fabry disease, Lyso-GB3: lysosomal globotriaosylceramide. ^a^ Abnormal findings of the GLA gene include class 3 variants (uncertain to be pathogenic), class 4 variants (likely to be pathogenic), and class 5 variants (certain pathogenic). ^b^ The diagnosis FD is confirmed in women when the abnormal findings of the GLA gene complemented with abnormal findings in the biochemical assessment (α-Gal A and Lyso-GB3).

In men, a normal α-Gal A activity and lyso-GB3 excretion excludes FD [14]. However, if the enzyme activity is reduced or the lyso-GB3 is increased, screening of the GLA gene for mutations is required to confirm FD (Fig 1A) [14, 31]. In women, a normal α-Gal A enzyme activity [15] and normal lyso-GB3 excretion does not rule out FD. Due to skewed X-inactivation [32], it is known that the clinical manifestations in heterozygous females may range from asymptomatic to severely affected subjects. As GLA is the only gene in which mutations are known to cause FD [33], screening of the GLA gene for mutations is the most reliable method of diagnosing the carrier state in females. Biochemical findings of α-Gal A and lyso-GB3 could serve as biomarkers of disease severity [29]. Therefore, α-Gal A activity, lyso-GB3 excretion, screening of the GLA gene for mutations were performed simultaneously in women (Fig 1B) [14, 34].

### Standard protocol approvals, registrations, and patient consents

The Maastricht UMC’s Medical Ethics Committee and Board of Directors approved this study. According to the Code of Conduct for the use of data in Health Research [35], for this type of retrospective study, informed consent does not need to be obtained if the data are used anonymously and patients are given the opportunity to object against the use of their medical and personal data for research (which is the case in the Maastricht UMC+). All data were obtained from medical records. Patient records were anonymized and de-identified prior to analysis. The individuals described in this manuscript have given written informed consent for publication of their case details.

## Results

### Patient selection and general characteristics

A total of 1040 patients were referred to the Maastricht UMC+ for possible SFN. In 771 patients (74%) the diagnosis SFN was confirmed. Forty-six patients with SFN were excluded from the study based on non-available or incomplete FD diagnostic data. Eventually, a total of 725 patients with SFN were screened for FD (305 (42.1%) men, 420 (57.9%) women). All patients except seven were adults (99%). The median age of onset of the complaints was 47, with a standard deviation of 14.3. Possible underlying causes in our patient cohort were diabetes mellitus (n = 35; 4.8%; 4 type 1 diabetes (0,6%), 31 type 2 diabetes (4,3%)), sarcoidosis (n = 23; 3.2%), morbus Sjögren (n = 11; 1.5%), monoclonal gammopathy of undetermined significance (n = 5; 0.7%), and hypothyreoidism (n = 3; 0,4%).

Besides redness of the hands or feet in 30.9% of the 725 patients, no other skin abnormalities were seen. None of the patients had a history of cardiomyopathy of renal failure.

### Diagnostics for Fabry disease

**Fig 2:**
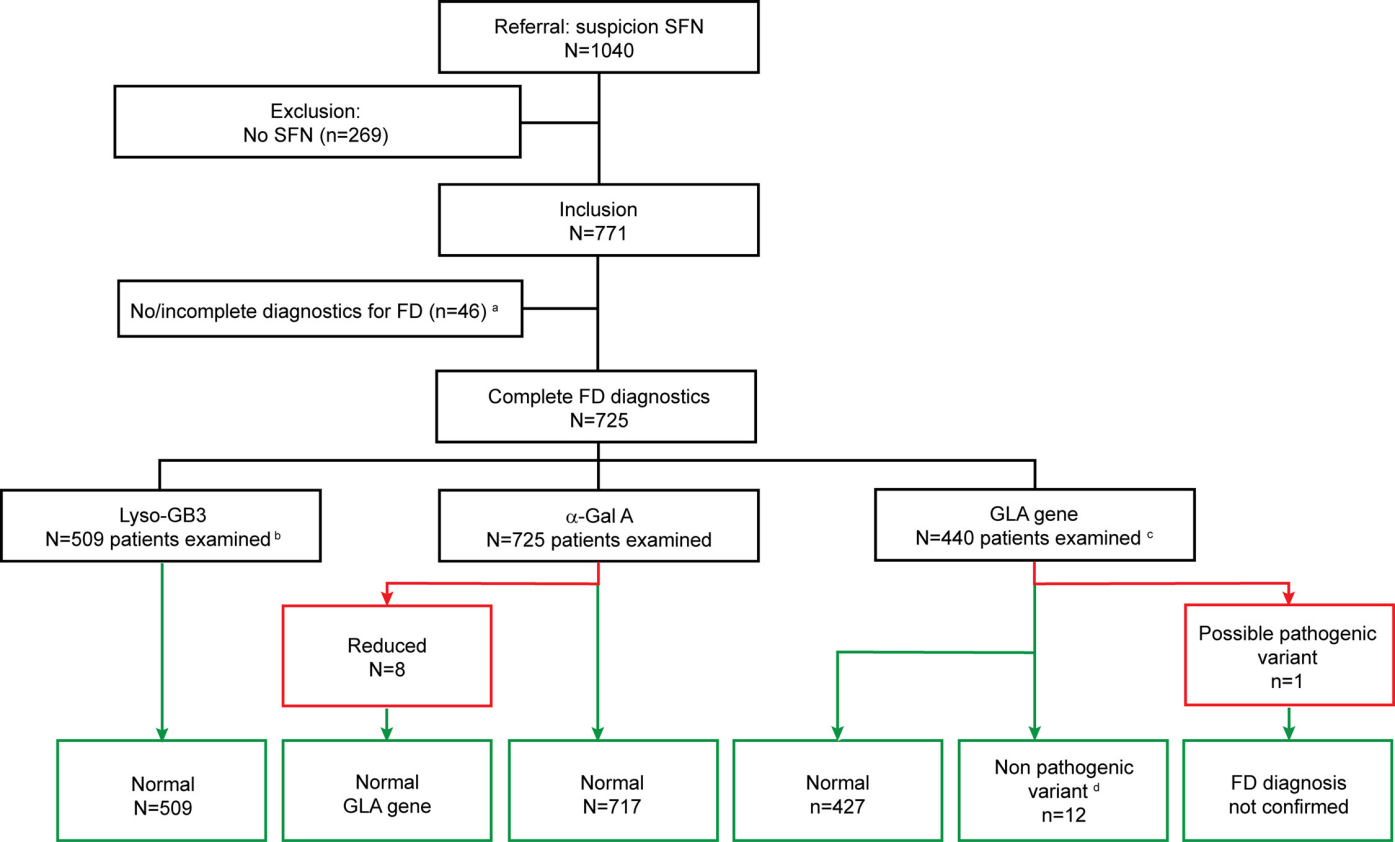
Small fiber neuropathy patients analyzed for Fabry disease in the Maastricht University Medical Center. Illustration of the outcome of investigations to confirm the diagnosis of Fabry disease. α-Gal A: α-galactosidase A, Lyso-GB3: lysosomal globotriaosylceramide, FD: Fabry disease, SFN: small fiber neuropathy. ^a^ Missing data. ^b^ The measurement of lyso-GB3 excretion in urine was incorporated in our workflow for SFN patients from April 2012. ^c^ GLA gene sequencing was performed in all women, and in males in case of reduced α-Gal A enzyme activity. ^d^ These includes the class 2 variants (unlikely to be pathogenic) and the class 3 variants (uncertain to be pathogenic).

The three tests for FD, α-Gal A activity in blood, lyso-GB3 excretion in urine, and GLA gene sequencing, were applied to the study population as presented in Fig 2.

#### α-Gal A enzyme activity

α-Gal A enzyme activity was determined in leukocytes of all 725 eligible patients. In eight patients (1.1%) a reduced α-Gal A enzyme activity was found, ranging from 2.8 to 26.9 mmol/L. The α-Gal A enzyme activity was normal in the other 718 patients (30.3–191.9 (mean 81.2) mmol/L).

#### Lyso-GB3 excretion in the urine

Five hundred nine of the 725 SFN patients were tested for lyso-GB3 excretion in the urine. All results were normal (100%). Five of these 509 patients with a normal lyso-GB3 excretion had a diminished α-Gal A enzyme activity (7.1–26.9 mmol/L).

#### GLA gene screening for possible mutations

A total of 440 patients underwent screening of the GLA gene for mutations. Thirteen SFN patients (3.0%) carried a variant in the GLA gene (Table 1).

**Table 1:** GLA gene variants and results of biochemical testing in a population of SFN patients (n = 725).

Patient	c.position variant	p.position variant	Class	α-Gal A (mmol/L)	Lyso-GB3 (nmol/mmol creatinine)
1 F	c.-40G>C	-	2	57.4	NA
2 F	c.-8C>G	-	2	68.8	NA
3 F	c.48T>G	p.Leu16Leu	2	96.7	NA
4 F	c.123C>T	p.Thr41Thr	2	72.9	NA
5 F	c.352C>T	p.Arg118Cys	4	59.2	0
6 F	c.376A>G	p.Ser126Gly	2	52.6	0
7 F	c.801+21T>C	-	2	43.2	0
8 F	c.937G>T	p.Asp313Tyr	3	62.4	NA
9 F	c.937G>T	p.Asp313Tyr	3	60.6	NA
10 M	c.937G>T	p.Asp313Tyr	3	30.3	0
11 F	c.937G>T	p.Asp313Tyr	3	51.5	0
12 F	c.937G>T	p.Asp313Tyr	3	63.5	0
13 F	c.999+11_12ins11+999+16_20del5	-	2	78.9	NA

α-Gal A: α-galactosidase A enzyme activity, F: female, Lyso-GB3: lysosomal globotriaosylceramide, M: male, NA: not available.

Of the nine variants identified [c.-40G>C; c.-8C>G; c.48T>G (p.Leu16Leu); c.123C>T (p.Thr41Thr); c.352C>T (p.Arg118Cys); c.376A>G (p.Ser126Gly); c.801+21T>C; c.937G>T (p.Asp313Tyr) (n = 5); c.999+11_12ins11+999+16_20del5], only one variant (c.352C>T (p.Arg118Cys) was classified as a likely pathogenic. The other eight variants were classified as a class 2 or class 3 variant. The eight patients with a reduced α-Gal A enzyme activity were also part of this cohort and showed no abnormalities.

The patient carrying the c.352C>T variant heterozygous was a 33-year old female. Her α-Gal A enzyme activity was normal (59.2 mmol/L). The patient was referred to a tertiary referral and treatment center for FD in the Netherlands, where a detailed work-up of potential FD-pathologies and biomarkers was performed. The α-Gal A enzyme activity was retested and normal (53.0 mmol/L). For this patient no excretion of lyso-GB3 was detected in urine. During three years of follow-up, the patient did not develop symptoms or signs of FD (skin lesions, cornea verticillata, cardiac or renal involvement). Therefore the diagnosis of FD could not be confirmed. All patients with a class 2 or 3 variant had a α-Gal A enzyme activity >30 mmol/L, and if tested, normal lyso-GB3.

In total, 725 patients underwent testing for FD, and the diagnosis FD was not confirmed in any of these patients.

### Diagnostics for FD costs

In our center, the costs per person for testing α-Gal A enzyme activity in leukocytes are $ 1,186, for Lyso-GB3 in urine $ 883, and for GLA gene sequencing $ 1,017, which is a total of $ 2,069 (for men) and $ 3,086 (for women) per patient. In this cohort of 725 patients the costs for FD diagnostics were $ 1,756,777.

## Discussion

In a large cohort of patients diagnosed with isolated SFN who were tested for FD (n = 725), we did not find any patients with FD. In only one patient a possible pathogenic variant was identified, but after thorough investigations and years of follow-up, the diagnosis FD could not be established. Because FD is a treatable disease, early diagnosis can prevent further complications of the disease and may improve the quality of life [17–20]. However, little is known about the prevalence of SFN as first symptom of FD. Previously, only two small cohort studies examined the presence of GLA gene mutations in idiopathic SFN. The first study identified one GLA gene mutation in a cohort of 24 patients diagnosed with idiopathic SFN [36]. In a second study, in one out of 29 patients with idiopathic SFN, a variant of the GLA gene was shown. As in our study, the diagnosis FD could not be made in any of these patients [37]. However, our study is the first that systematically examined the prevalence of FD in a large clinically well-defined cohort of patients diagnosed with isolated SFN.

Our results show isolated SFN not being the first and certainly not the only symptom of FD. Patients with FD probably also have other symptoms, like skin lesions or hearing loss, cardiac or renal involvement leading to the diagnosis of FD [17]. These symptoms could be accompanied with neuropathic pain, but the neuropathic pain alone does not seem typical for adult FD patients. A previous study showed that only 12 patients, out of a cohort of 366 patients with FD, had neurological signs or symptoms, without the involvement of any other organs [15]. The general view at our center is that patients with potential SFN related complaints are being referred and examined, often without an underlying etiology. Possible referral bias is conceivable in cases having other symptoms indicative for FD being referred to a tertiary center for FD.

Neuropathic pain has been addressed as one of the first symptoms of FD in some cases [11–13, 16]. However, most of these studies concerned children, adolescents and young adults [17, 38]. These observations were not confirmed by the current study in adults with isolated SFN. In contrast with literature [13, 15], none of the children in our study cohort were diagnosed having FD. However, some caution is warranted since only 7 children were part of the study population.

From the current results, it is clear that the suspicion of FD is extremely low, if not absent in adult patients presenting with SFN without concomitant symptoms or signs compatible with FD (skin lesions, hearing loss, cardiac or renal involvement). In addition, genetic and biochemical testing for FD is expensive, more than 1.7 million US dollars in the cohort (n = 725) examined in the current study with no patient being diagnosed having FD. Therefore, in adult patients with isolated SFN, routine screening for FD does not seem warranted.

In case testing for FD is warranted, we would recommend to start with the GLA gene sequencing as the first test [14, 39, 40]. If this test turned out to be normal, the patient is diagnosed not having FD. Whenever a variant of the GLA gene is found, the diagnostics could be complemented with the α-Gal A enzyme activity, and other biochemical assessments necessary, to confirm the diagnosis FD. This strategy would largely reduce the costs of diagnostic testing for FD.

## Supporting Information

S1 Dataset This is the dataset of all the data used for this manuscript. (XLSX)

S1 Form This is an example of the informed consent that is given by the patients included in this manuscript. (PDF)
