# Supplementary material for: No Fabry Disease in Patients Presenting with Isolated Small Fiber Neuropathy
Source: PLoS One. 2016 Feb 11;11(2):e0148316. doi: 10.1371/journal.pone.0148316 (PMC4750945; doi:10.1371/journal.pone.0148316)
Supplement: S1 Form — (PDF) [file pone.0148316.s002.pdf]

Consent form

For a patient's consent to publication of information about them in a scientific journal

Name of patient:____
Subject matter publication:____
Authors*:____
____
*it is possible that additional authors are added

I____ [insert full name] give my consent for this information about myself relating to the subject matter above to appear in a journal and associated publications.*

I understand the following:
(1) The Information will be published without my name attached and the authors will make every attempt to ensure my anonymity. I understand, however, that complete anonymity cannot be guaranteed. It is possible that somebody somewhere - perhaps, for example, somebody who looked after me if I was in hospital or a relative - may identify me.
(2) The Information may be published in the journal, which is distributed worldwide. The journal goes mainly to doctors but is seen by many non-doctors, including journalists.
(3) The Information will also be placed on a journal website
(4) The information will not be used for advertising or will not be used out of context.
(5) I can revoke my consent at any time before publication, but once the Information has been committed to publication ("gone to press") it will not be possible to revoke the consent.

Signed:_________________________________ Date: ______________________
